# Drug self-assembly for synthesis of highly-loaded antimicrobial drug-silica particles

**DOI:** 10.1038/s41598-018-19166-8

**Published:** 2018-01-17

**Authors:** Cameron A. Stewart, Yoav Finer, Benjamin D. Hatton

**Affiliations:** 1 Institute of Biomaterials and Biomedical Engineering, University of Toronto, Toronto, Ontario Canada; 2 Faculty of Dentistry, University of Toronto, Toronto, Ontario Canada; 3 Department of Materials Science and Engineering, University of Toronto, Toronto, Ontario Canada

## Abstract

Antimicrobial drug release from biomaterials for orthopedic repair and dental restorations can prevent biofilm growth and caries formation. Carriers for drug incorporation would benefit from long-term drug storage, controlled release, and structural stability. Mesoporous silica, synthesized through a co-assembly of silica and surfactant template, is an ideal drug encapsulation scaffold that maintains structural integrity upon release. However, conventional loading of drug within meso-silica pores via concentration-gradient diffusion limits the overall payload, concentration uniformity, and drug release control. Herein we demonstrate the co-assembly of an antimicrobial drug (octenidine dihydrochloride, OCT), and silica, to form highly-loaded (35% wt.) OCT-silica nanocomposite spheres of 500 nm diameter. Drug release significantly outlasted conventional OCT-loaded mesoporous silica, closely fit Higuchi models of diffusive release, and was visualized via electron microscopy. Extension of this concept to the broad collection of self-assembling drugs grants biomedical community a powerful tool for synthesizing drug-loaded inorganic nanomaterials from the bottom-up.

## Introduction

Engineering vehicles for highly localized, targeted drug delivery has become a significant requirement of many new therapeutic treatments to target tumors or bacterial infection, due to the systemic toxicity, resistance development, and undesirable side effects that many effective therapeutics pose^1,2^. Antibiotic drug release in a localized environment can target a bacterial infection at an efficacious local concentration, above the minimum inhibitory concentration (MIC), while reducing risk of systemic toxicity and antibiotic resistance development^3–5^.

Recurrent caries, known also as secondary caries, at the margins of dental restorations is the result of acid production by caries-causing bacteria that reside in the restoration-tooth interface is a major reason for dental restorative materials’ (fillings) failure, and may affect over 100 million patients a year in the US, at a cost of $34 billion^6–11^. In addition to acid production, caries-forming bacteria have enzymatic activity that degrades the materials and the restoration-tooth interface and may contribute to the formation and progression of recurrent caries^9,10^. To mitigate the above processes, there has been on-going interest in introducing local antimicrobial chemotherapy at the restoration to prevent bacterial proliferation and caries formation^12,13^ since it is impractical to treat the local infection with systemic antimicrobial delivery because of limited drug accessibility to the site of infections that require high systemic doses^10,14^. In particular, we anticipate that antimicrobial particles included into the adhesive layer of dental fillings, applied at the restoration-tooth margins, could be designed to release antimicrobial drug in this local environment to delay biofilm progress over a clinically relevant timescale of years^15^.

An important factor for drug encapsulation particles to achieve efficient targeted and controllable drug release is high drug loading (volume % concentration) to maintain an effective concentration of released drug over long timescales since when drug reservoir is limited, the mass of released drug will also be limited at any given time as it is directly proportional to the total reservoir of drug^16^. In a biological analogy, secretory vesicles such as pancreatic insulin granules or platelet alpha-granules feature dense, high concentration packing of enzyme or growth factors in highly-organized volumes, which are then triggered to release their contents^17^. Synthetically, the high concentration loading of drug molecules into nanomaterials remains a challenge because it requires some degree of self-organization^18^. Degradable drug storage and release vectors such as liposomes, nano-gels, micelles and layer-by-layer systems can achieve a wide range of drug loading (from <1% to over 50%)^19–23^ for targeted drug delivery, and programmed release for tumor targeting and insulin release, with their assembly driven by a range of intermolecular forces and chemical reactions^24^. Lysosome-inspired polymersomes, for example, have been shown to sequester anticancer drug molecules at up to a 1:1 ratio of polymer to drug^25^. In another study, granule-like colloids of the drug fulvestrant, stabilized with targeting proteins, acted as their own nanoparticle without the use of a third encapsulating material^26^.

Drug-loaded particles for orthopedic implant coatings or dental restorative materials should also ideally have an inert, nondegradable scaffold which can maintain structural integrity following release of the drug so not to compromise the implantable device or restorations, and have predictable drug release rates^4^. Progress has been made in addressing these challenges for bone scaffold materials in addition to being osteoconductive and angiogenic, but long-term, predictable, and engineered release of antimicrobial from these materials remains difficult^27^. Highly ordered nanoporous inorganic scaffolds, such as mesoporous silica nanoparticles (MSNs), are ideal because they have ordered, identical channels (2–3 nm diameter), are structurally and chemically stable, biocompatible, highly porous (50 volume %), and remain intact after drug release^28–31^. MSNs are better suited than conventional sol-gel silica (or other inorganic) drug reservoirs, where pores are frequently not interconnected and the scaffold structure often degrades upon release^32^.

**Figure 1:**
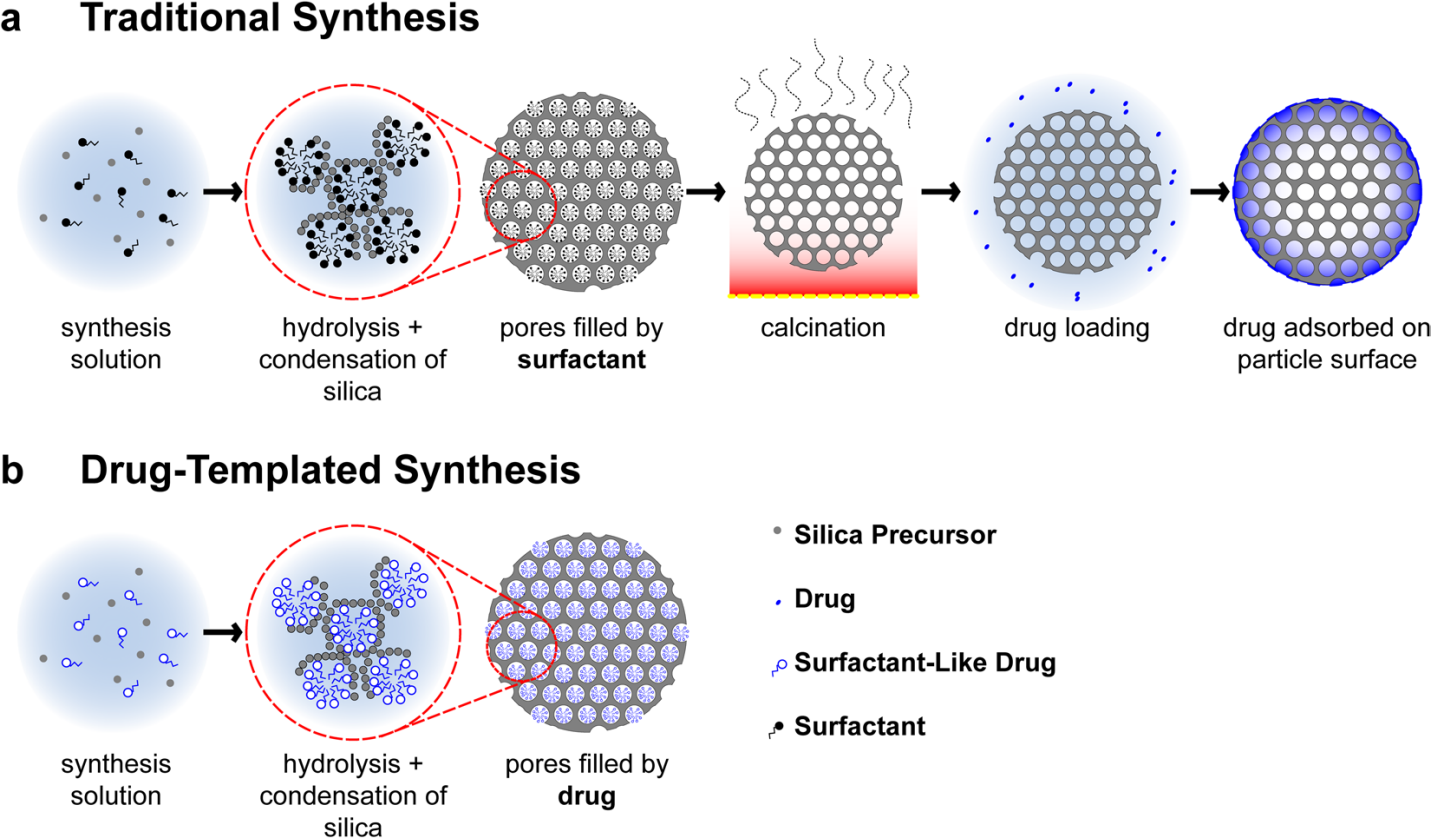
Drug-templated synthesis of MSNs (**b**) significantly simplifies synthesis and yields vastly different drug-loading and release results when compared to traditional concentration-gradient driven loading (**a**).

Examples of MSNs loaded with drugs for diffusional release include antimicrobials such as chlorhexidine, peptides, and biosurfactants^16,33,34^. Ordered mesoporous silica, often with hexagonally-packed channels, is formed through the condensation of a silica precursor in co-assembly with an organic surfactant template to form a porous mesostructure following the removal of the template (Fig. 1a)^28,31,35–37^. MSNs have sufficient mechanical strength for antimicrobial applications as filler particles for composites^38^, or as coatings by ‘evaporation-induced self-assembly’ deposition^16^. The templating agent may be a molecule that forms micelles and presents a hydrophilic interface for silica to condense around, such as surfactants and block co-polymers^39,40^.

Nanoporous inorganic scaffolds such as MSNs can be loaded by long-term suspension in a drug solution through diffusion, through the evaporation of a drug solution (incipient wetness technique), or through suspension in a melted liquid drug, when the drug chemistry will allow, for example with ibuprofen^41^. Typically, a concentration gradient is used to drive drug diffusion into the pores, and results in internal loading of <1% wt. The pore infiltration by a drug-carrying solvent is inherently limited by the drug solubility^16,35,42–45^. Alternatively, macromolecular layers and surface modifications are used to attract and trap drug molecules^30,46^. For example, loading the poorly-soluble anticancer drug camptothecin into 70% porous MSN, the 1.7 mg ml^−1^ drug-dimethyl sulfoxide solution will give a theoretical maximal loading of 0.2% wt. drug (approximately 0.05% wt was achieved)^35^. Even a saturated (200 mg mL^−1^) solution of the highly soluble antiseptic chlorhexidine gluconate in water^47^ would only result in a theoretical maximum 18% wt. drug in MSNs. Insipient wetness loading forces crystallization of 100% of drug in solution within and around MSN particles^41,48^, but it typically forms crystallized drug on the particle exterior, which is then “released” via rapid dissolution and first-order kinetics^49,50^. Loading by melting similarly results in drug crystalizing in the particle interior and exterior^51^, and is only suitable for drugs that do not decompose before melting^4^.

We have developed a bio-inspired alternative to post-synthesis MSN drug loading, to self-assemble an antimicrobial drug (octenidine dihydrochloride, OCT) directly with silica to form an ordered drug/silica nanocomposite (mesostructure) (Fig. 1b). OCT is a cationic surfactant antiseptic that shows broad efficacy against gram positive and negative bacteria^52^. OCT has high biocompatibility, no known bacterial resistance, and is used currently as a mouth rinse, wound cleansing agent, topical antiseptic, and in other applications^52–54^. The self-assembly and micellar properties of OCT have not previously been investigated.

While there have been attempts to modify model drugs to be used as amphiphilic templates^55^, as well as using non-drug agents for templating mesostructured particles that release upon degradation of the particles^34^, this work represents the first example of a structurally-stable drug-MSNs for an existing, commercially available drug. Through incorporation with dental restorative adhesives or applied as an implant coating, these OCT/silica MSNs can target bacteria in the confined volumes of degraded restoration-tooth interface, where cariogenic bacteria reside^10^ and over implant surfaces. We hypothesize that OCT-templated silica mesostructured particles will contain significantly higher drug loading when compared with traditionally synthesized particles, and therefore could provide efficient, long term antimicrobial release from composite polymer materials and implant surfaces.

## Results

### OCT-MSN Physical analysis

**Figure 2:**
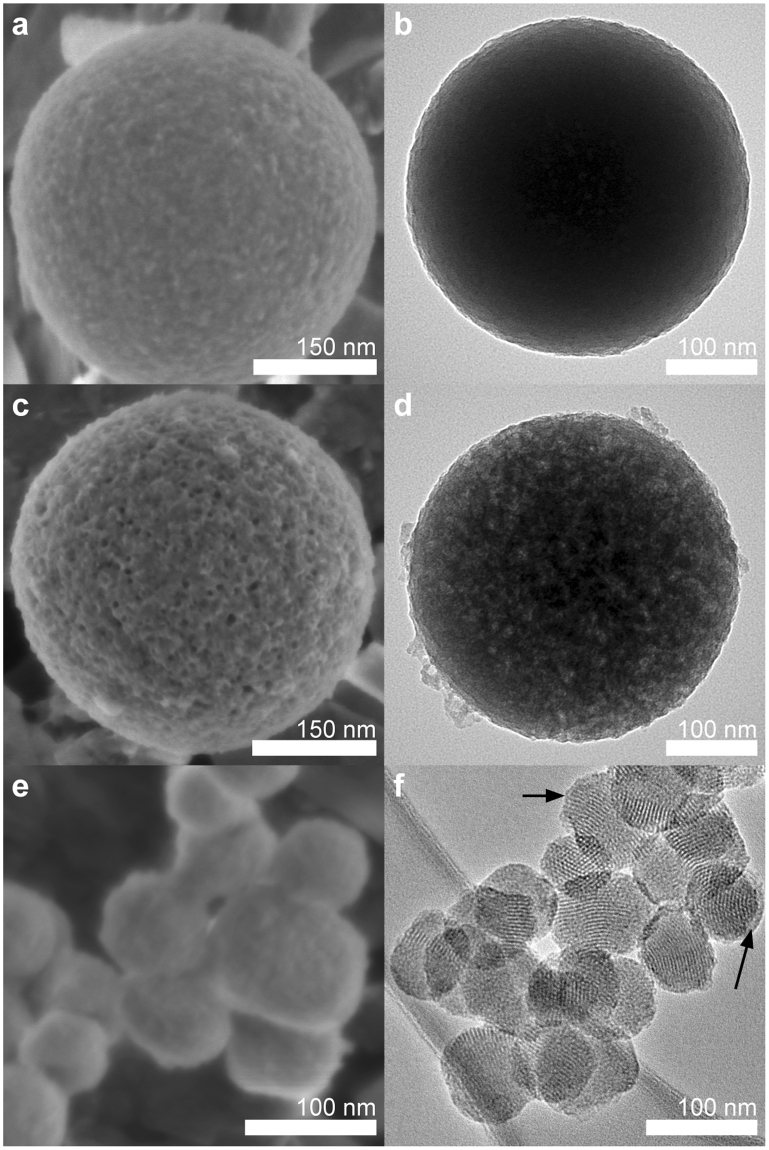
Microscopy of OCT-MSNs and control particles contrast the results of drug-templating and diffusion gradient drug loading. SEM and TEM micrographs of drug-templated MSNs before drug release (**a** and **b**, respectively) show well defined and separated particles approximately 424 nm in diameter. Post-drug-release OCT-MSNs (SEM in **c**, TEM in **d**) show very clear surface pore openings and well resolved pores throughout the particle. Control OCT-loaded MCM-41 (SEM in **e**, TEM in **f**) exhibits fusing of particles and a layer of external drug (highlighted with arrows).

We measured the critical micelle concentration (CMC) of OCT to be 3.79 mM by monitoring conductivity change as drug concentration was lowered. For comparison, the CMC of cetyltrimethylammonium is 0.90 mM^56^. A precipitation-based synthesis of drug-templated particles (OCT-MSNs) was carried out at varying concentrations of OCT in a basic aqueous solution using tetraethyl orthosilicate (TEOS) as a silica source. Traditional MCM-41 MSNs were also synthesised as a control, using cetyltrimethylammonium bromide (CTAB) as a pore templating agent, which was subsequently removed via 3 post-synthesis washes and calcination^37^. Loading of MCM-41 control was carried out through a solvent-evaporation process for a theoretical 40% wt. OCT in the particles. Scanning electron microscopy (SEM) showed the OCT-MSNs to be spherical particles with relatively high size monodispersity and average diameter of 424 ± 75 nm (high magnification of single particle in Fig. 2(a), example overview of population of particles used for measurement shown in Supplementary Figure S2). The as-prepared drug-loaded particles appear to have a slightly dimpled texture with no visible porosity. Transmission electron microscopy (TEM) showed the OCT-MSNs to appear solid, with little internal contrast immediately post-synthesis (Fig. 2b).

In Fig. 2(c), OCT-MSNs are shown after sonication in ethanol for 2 hours, with a rough, porous structure exposed, suggesting drug removal from the particles exposes the underlying porous silica. This drug-removal mechanism is corroborated by drug release detection in the ethanol using UV-vis and TGA of OCT-MSNs post-ethanol-sonication. In Fig. 2(d), a disordered porous structure is visible in all particles in samples that have had drug removed by ethanol sonication, due to increased contrast between silica and vacuum compared to silica and drug. These post-drug-release particles also appear identical in size and shape to as-synthesized particles, indicating that drug release is not dependent on degradation of the silica scaffold as for other sol-gel systems^57^, but rather by diffusion from pores.

SEM and TEM of OCT-loaded MCM-41 (Fig. 2e and f respectively) shows 71 ± 9 nm diameter particles with a highly ordered hexagonally packed porous structure. SEM images appear to show particles fused together by material. TEM images show a layer of material deposited on the exterior of these particles beyond the hexagonally packed pore structure. Since particles were washed and calcined prior to evaporative drug loading, it is presumed that this external material is drug crystalized on the surface of particles. This is in contrast with the uncoagulated OCT-MSNs that do not appear to possess this external layer.

**Figure 3:**
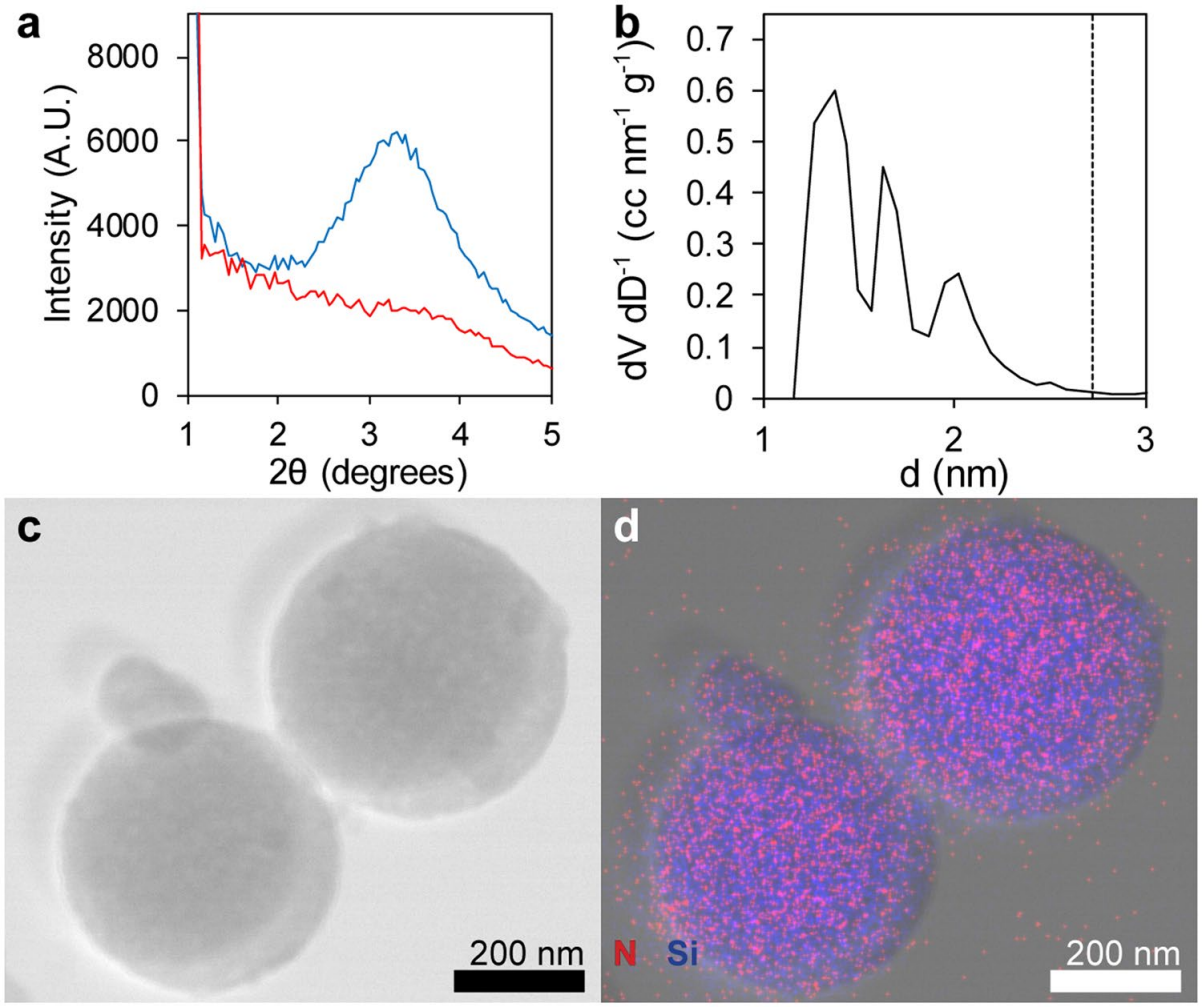
Physical characterization of OCT-MSNs demonstrates the relationship between templating drug molecule and MSN structure. The displayed XRD results (**a**) contain a low, broad diffraction peak typical of as synthesized OCT-templated MSNs (blue), as well as the lack of peak in particles synthesized below the OCT critical micelle concentration (red). DFT analysis (**b**) of N_2_ adsorption data shows 3 pore diameters (▬) approximately ½ the XRD d-spacing (▪▪▪). Scanning TEM (**c**) and overlaid EDX map (**d**) of OCT-MSNs confirms the presence of OCT with particles: Si signal emanates from the silica particles, while N (unique to the loaded OCT) signal is confined to the area of MSNs.

Low angle powder X-ray diffraction (XRD) in Fig. 3(a) of OCT-MSNs reveals a broad diffraction peak corresponding to a d-spacing of 2.64 ± 0.11 nm, indicating a semi-ordered porous structure^16,35,58–60^. Interestingly, the diffraction peak shifts little as OCT concentration changes in the synthesis solution, but disappears completely when the concentration is brought below its CMC. These results clearly demonstrate that OCT micelle self-assembly is contributing to a mesophase formation. The XRD peak also remains after drug removal via sample calcination (550 °C in air), with d-spacing decreasing 0.23 ± 0.03 nm, further indicating that diffraction is due to a templated silica mesostructure and not due to crystalized drug. Shrinking d-spacing of about 10% is the result of increased silica condensation and densification at higher temperature and is expected for mesoporous silica^40,61^. The lack of a sharp XRD peak indicates the absence of a well-ordered mesophase. OCT, as a di-surfactant, may not favor assembly into a rigid hexagonally packed assembly of rods but rather a rapidly changing worm-like array of extended micelles^62^. The XRD spectra is consistent with other worm-like structures where disordered pores run continuously throughout the MSNs^19,56^.

Brunauer-Emmett-Teller (BET) analysis by N_2_ adsorption/desorption was used to determine pore size, volume, and total surface area^39,40,56^. Surface area for calcined OCT templated MSNs was found to be 856 m^2^ g^−1^ which is lower but comparable to that of the MCM-41 control (1098 m^2^ g^−1^) and other mesoporous materials (the theoretical value for smooth spheres of an equivalent size and density is just 13.1 m^2^ g^−1^). The pore volume was 0.47 cm^3^ g^−1^, or approximately 50.8% porosity, compared to 1.084 cm^3^ g^−1^ (70.4% porosity) for the MCM-41. Lower pore volume may be attributed to the less ordered mesophase of OCT-MSNs when compared to hexagonally closed-packed MCM-41 pores. The high open porosity of as-synthesized MCM-41 supports the notion that externally deposited material is drug condensed on the particle surface upon loading, and not material previously deposited during synthesis or template removal.

Density functional theory (DFT) analysis of BET isotherms in Fig. 3(b) shows that OCT-MSNs have a trimodal pore size distribution of approximately 1.4, 1.7 and 2.0 nm diameter, suggesting the presence of multiple mesophases within particles, helping account for prior “disordered” XRD results. The “tail-head-tail-head-tail” arrangement of OCT, whose corresponding theoretical straight tail lengths are approximately 1 nm, may allow for a broad range of conformations^62^ to maximize hydrophobic/hydrophilic and electrostatic interactions under dilute conditions^63,64^, and thus a distribution of pore diameters may be expected. The d-spacing is approximately twice the average pore size, suggesting an approximate 1:1 ratio of drug-filled pore and silica structure when viewed in cross-section, which corroborates the 50% porosity by BET analysis.

OCT-loading was determined by thermogravimetric analysis (TGA) to be 34.4 ± 1.4% wt. This drug content is clearly significantly higher than conventional drug-loaded MSN having <1% wt. internal loading^16,35,42–45^. Post-synthesis loading of drug in tortuous pore structures like those seen here is usually much lower than that of MCM-41 due to the longer diffusion path^41^. This long diffusion path also affects release, slowing the movement of drug from deep in the particles outward. The high-loading and high-tortuosity of OCT-MSNs should both contribute to a greatly increased release timeframe. As discussed in the introduction, evaporation techniques may still claim higher degrees of loading, but a large amount of the drug is incorporated in the product as separate crystals or on the surface of MSNs^41^, which was confirmed in microscopy of the MCM-41 control (Fig. 2e,f). We have confirmed using mass spectrometry the identity of the released molecule, from OCT-MSNs stored for 21 months after synthesis, as pure OCT that is identical to the compound used for synthesis.

Energy dispersive x-ray (EDX) mapping was performed for Si and N to determine the spatial distribution of silica and the OCT phases (Fig. 3c,d). Very strong N signal was obtained from particles that were otherwise free of external debris or visible coating layer, providing evidence that the OCT micelles interpenetrate the silica network as a uniform mesostructure.

### Drug Release Kinetics of OCT-MSNs

**Figure 4:**
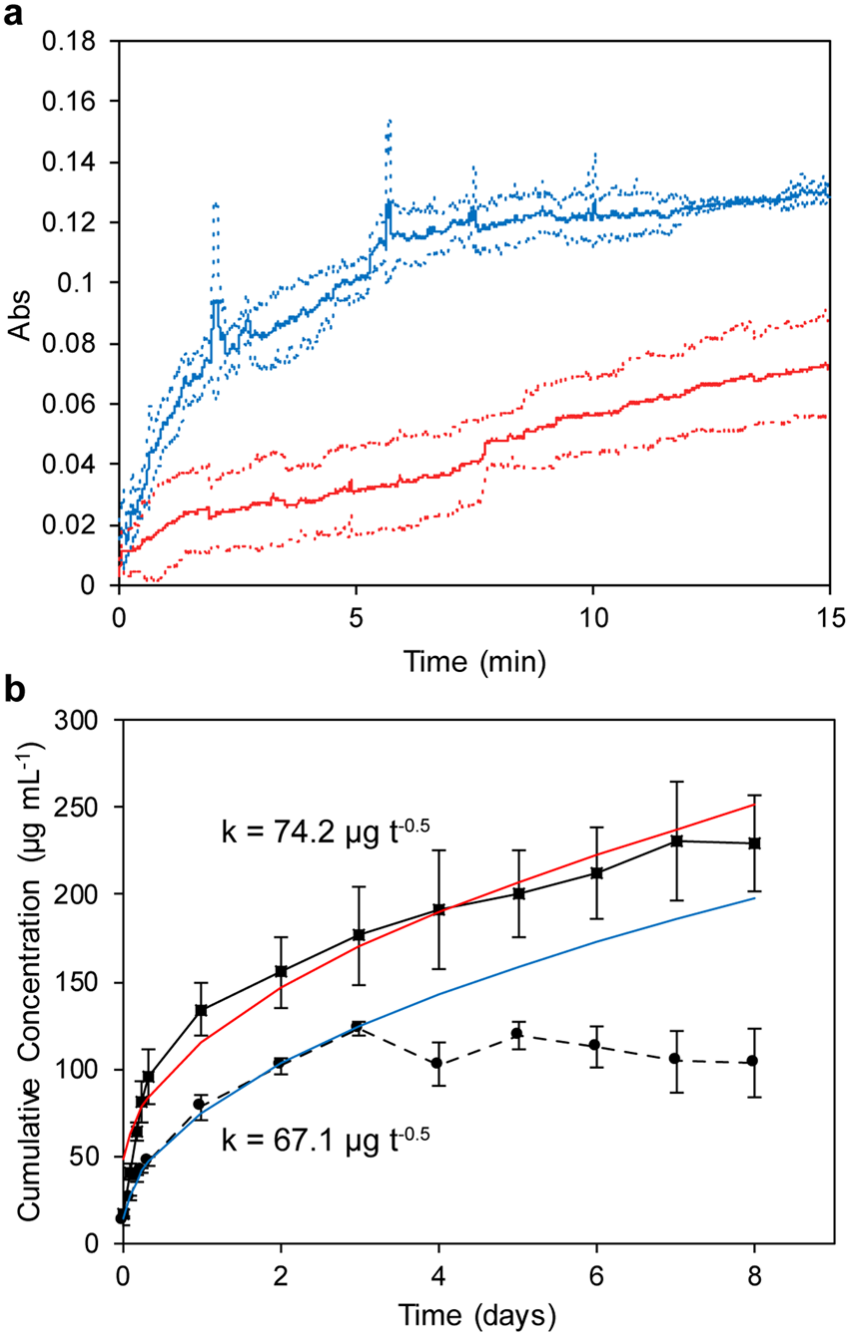
OCT-MSNs release drug more slowly and more predictably than OCT-loaded MCM-41. (**a**) Absorbance of OCT at 281 nm, measured by fibre optic probe, released from either control OCT-loaded MCM-41 (blue) or OCT-MSNs (red). (**b**) Cumulative release from OCT-MSNs (▪, ▬) compared to OCT-loaded MCM-41 (●, ▪▪▪). Higuchi model kinetic parameters k are displayed alongside fitted modeled release from OCT-MSNs (red) and OCT-loaded MCM-41 (blue). Error dotted lines (**a**) or bars (**b**) represent ± 1 standard deviation, N = 3.

Short-term drug release from OCT-MSNs or MCM-41 control at 0.02 mg mL^−1^ of particles suspended in phosphate buffered saline (PBS) (pH = 7.2, 22 °C) was monitored by UV-Vis absorption at 281 nm and compared with release from the MCM-41 control (Fig. 4a). Due to the difference in size between OCT-MSNs and MCM-41 control, the rate of the release alone is not a sufficient means of comparison, and we therefore must investigate the mechanism and model of release. Release from MSN pores has been previously found to follow the kinetics described by Higuchi for a spherical particle with a granular matrix containing a soluble compound, and may be approximated by the formula for release from a granular matrix plane for release below 50% of total drug loading^45,49,55^. In this model, cumulative release (Q) over time (t) is given by

1 $$Q=A\ast f({\rm{D}},\varepsilon ,\tau ,{\rm{S}},{{\rm{C}}}_{{\rm{s}}})\ast {t}^{0.5}$$

where A is the fixed spherical outer surface area of the silica particles, D is the diffusivity of drug in the solvent through matrix pores, ε is the porosity of the matrix (silica without templating drug), τ is a tortuosity factor to account for an increased diffusion path in non-linear pores, S is the solubility of the drug in the release media, and C_S_ is the mass of drug per unit volume of matrix. These parameters may be simplified to k (units of µg t^−0.5^). Release from OCT-MSNs fits well to a t^0.5^ profile (R^2^ = 0.94), while release from OCT-MCM-41 deviates (R^2^ = 0.88) (supplemental material). The release of drug from MCM-41 has a release profile inconsistent with the Higuchi model during initial exposure to solvent which we suggest is due to the dissolution of the drug crystallized externally on and between clusters of particles, seen previously in SEM (Fig. 2e,f). Because OCT is confined to the pores of the OCT-MSNs, its release is limited by diffusion of solvent into pores and subsequent diffusion of drug out of the particle. This internal confinement avoids the initial uncontrolled burst of drug release typically seen with highly-loaded traditional mesoporous materials^65^.

Release from OCT-MSNs and the MCM-41 control was monitored over an 8-day period, as shown in Fig. 4(b) with their calculated k parameters for release kinetics. Samples were suspended in a fixed 10 mL volume of (PBS) at 2 mg mL^−1^, 37 °C and at a pH of 7.2. Continuous release of drug from OCT-MSNs corresponding to 34% initial loading was observed after 8 days. Fitting release to the Higuchi model yielded a rate of 0.43 µg cm^−2^ d^−0.5^ (R^2^ = 0.96) and a theoretical t^1/2^ of 21.5 days. The tight drug packing within OCT-MSN pores and size-similarity between pores and the OCT molecule maximizes drug-silica interactions and lowers the rate of diffusive release from particles. The diffusion path of drug increases deeper into the particle, and release rate slows over time. Most importantly, release may be accurately modeled and predicted based on surface area and therefore particle size using the Higuchi formula thanks to consistent drug loading throughout the silica porous matrix resulting in one consistent phase of release. In the control, after the initial burst seen previously, release from MCM-41 quickly plateaus and stops when the loosely-bound crystalized drug is released from the particle surface and between particles as well as pores near the particle surface. When the control is modeled with the Higuchi equation over the first 3 days, release is only 0.043 µg cm^−2^ d^−0.5^ (R^2^ = 0.99) due to the theoretical high number of smaller particles and therefore high specific surface area. From SEM results in Fig. 2(e) it is clear that particles are largely fused together by drug, affecting the actual exposed surface area. Release will proceed in an uncontrolled manner that begins with dissolution of drug from the outside of these agglomerates in the first phase, but then changes as the agglomerates break apart and the surface area increases, exposing drug within the particle pores, effectively entering a second phase of release. The Higuchi model relies heavily on an unchanging surface area for modeling release, and is thus unsuitable for these externally loaded and agglomerated particles. Release stops after only 3 days and never reaches the theoretically loaded amount, suggesting inconsistent loading via evaporation within the sample (total loading from release data is 6% wt., far below 40% wt. target) possibly caused by the adsorption of drug onto the reaction vessel walls used for loading.

### Micrographic Tracking of Drug Release

**Figure 5:**
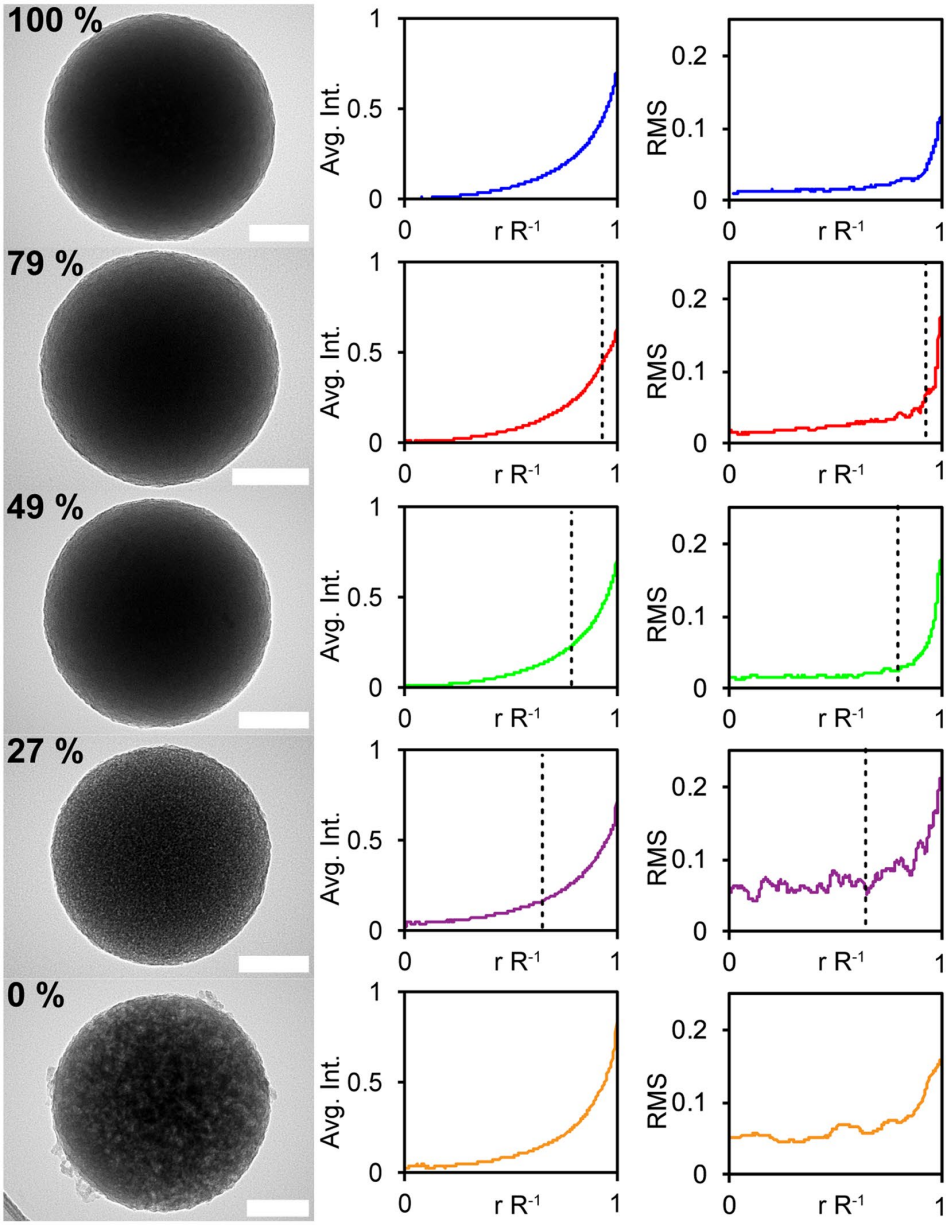
Particle porosity micrograph appearance is directly related to the proportion of drug released. Representative TEM micrographs of 100% through 0% loaded OCT-MSNs are shown on the left (scale bars are 100 nm). Normalized average radial intensity and normalized radial intensity RMS error (N = 7) is plotted beside each representative micrograph with the theoretical unextracted radius r’ marked by a dotted line.

TEM micrographs of particles at various stages of an accelerated release, performed by sonication in ethanol, are shown in Fig. 5 (left). The particles part-way through release are visually dissimilar, with gradual changes of appearance that resemble those differences observed between the fully-loaded particle in Fig. 2(b) and the empty particle following the drug’s release in Fig. 2(d). This contrast is difficult to discern visually, but is most stark between 100%, 27%, and 0% loaded particles. An image analysis process was sought to quantitatively describe these differences and compare populations of particles.

Although drug release from a spherical particle may be modeled as release from a plane up to 50% (as before), the more accurate Higuchi modeling for release from a sphere is described by the relation between *r*′ and *R*, the unextracted radius of the sphere and the initial unchanging matrix radius, respectively^49^. The relationship between these values and the residual drug fraction in the sphere (Re) is

2 $${\rm{Re}}={(\frac{r^{\prime} }{R})}^{3}$$

To estimate the amount of diffusional drug release, we have analyzed the relative intensity of TEM images of particles at different time points in solution. Total drug release and remaining content were analysed via UV-Vis and TGA, respectively, with both sets of results totalling the expected loaded amount for every stage of release (0 to 100%) within a ± 4% maximum margin of error. From Fig. 2, we expect the extracted outer shell to appear more porous than the inner drug-containing sphere, with greater variation of light and dark areas. Taking the radially integrated average of intensities in each particle image (Fig. 5 centre column) at every radii from the particle centre to edge does not capture this variation, as the intensity of light and dark areas is averaged around the circumference of each respective radii by the algorithm. The result displayed in Fig. 5 centre column shows the smoothing effect of this method, where the displayed intensity line is essentially just a function of the particle thickness at r R^−1^ and fails to capture any variation between drug-containing and drug-extracted regions. Instead, the total RMS error between the radially integrated average at each point along the radius and the intensity at each point along 2 random lines from the particle centre outward was taken for a total of N = 7 particles for each of the 6 loading samples, with pixel intensity normalized to the carbon-film background and a small point at each particle’s center at each stage of release (100% drug content to fully extracted). Areas of overlap, or with an inconsistent background, were excluded from analysis. These RMS values are plotted against the normalized radius (r R^−1^) and are presented in Fig. 5 (right) next to their corresponding particle loading and a representative image. Theoretical *r*′ values are displayed in each graph as a vertical dotted line. Although the outermost edge of even 100% loaded particles appears somewhat porous with a higher RMS error value, RMS errors appear to increase more rapidly starting at *r = r*′, indicating the beginning of a more porous-appearing region. This edge “porous” appearance may be due to the diffusive release of drug from pores starting at the particle surface and moving towards the centre. The very high uniformity of drug loading throughout these particles is what enables TEM intensity analysis of drug release from an individual particle, which is uncommon. The silica structure remains intact post-release, and release is not dependent and not a result of breakdown of the silica carrier. This observable adherence to the release model of individual particles provides an interesting illustration of the Higuchi model not previously reported on, and warrants further investigation.

### Retention of Activity of Released Antimicrobial

OCT is a well established effective broad-spectrum antimicrobial with a high degree of biocompatibility and no known formation of bacterial resistance^52,66^. However, analysis was performed to ensure that the stability and efficacy of OCT was preserved through the OCT-MSN synthesis process, and that released drug still functioned as an antimicrobial agent. Mass spectrometry was used to compare as-received OCT purity with OCT released from OCT-MSNs over 21 months under ambient conditions, with no difference in drug compound seen. Minimum inhibitory concentration (MIC) of as-received OCT and OCT-MSN-released OCT taken from the supernatant of the 8-day experiment above was performed at multiple concentrations against the cariogenic oral bacteria *Streptococcus mutans* UA159^67^. MIC was measured to be 2 µg mL^−1^ in both cases, in agreement with previous studies (between 1 and 32 µg mL^−1^ against a variety of pathogenic species in planktonic form) and further confirming that OCT was unchanged by the MSN synthesis process and will remain an effective antimicrobial agent^66,68,69^.

## Discussion

We have demonstrated that the drug-templated silica self-assembly produced particles with significantly higher loading of antimicrobial drug that traditionally synthesized particles, and therefore the hypothesis of the study that OCT-templated silica mesostructured particles will contain significantly higher drug loading when compared with traditionally synthesized particles is accepted. This drug/silica mesostructure is inherently loaded to a maximal drug content in its synthesis, as a highly-ordered micellar packing of drug molecules that does not require post-synthesis loading step. The release of the OCT drug via diffusion from pores is slowed by maximizing drug-silica intermolecular interactions due to the size matching between pore and drug micelle diameters. This release is more sustained and highly predictable than typical gradient-diffusion loaded porous materials. This idealized loading and release within porous silica enabled visual tracking of the drug release from the edge of the particle moving inwards, a visual analysis not normally possible with mesoporous silica loaded with drug post-synthesis. Besides more granular control of the final product, the synthesis demonstrated here is also considerably simpler and faster than conventional 2-stage MSN synthesis and loading, which increases the attractiveness of these particles as economical and easily manufactured controlled release vector. The results also offer an idealized example of the Higuchi model not normally seen in drug-eluting mesoporous silica materials. The drug retains its initial efficacy after synthesis and subsequent release against a common cariogenic bacterial species.

In these co-assembled materials, release rates are easily modeled due to the idealized drug loading within pores and lack of silica matrix breakdown during and after release, differentiating this process from a more typical amorphous sol-gel approach^32^. Other drugs exist with self-assembling and self-aggregating properties which could now be considered for mesoscale co-assembly to design a range of new drug encapsulation vehicles through bottom-up synthesis^70–72^.

Researchers have taken advantage of self-assembly of therapeutics and supporting carrier molecules, mimicking biological processes, to increase drug loading in carriers for some time, especially in the field of anticancer drug delivery^18,20,73^. These systems utilize intermolecular forces that cause components to arrange themselves in a predictable and repeatable manor, letting the drug delivery properties of the material be tuned to a high degree. We have taken this approach and expanded it to organic/inorganic co-assembly, introducing the benefits of inorganic systems unavailable from a strictly organic molecule approach. Previous work has shown the potential benefits of releasing the templating molecule in terms of kinetics and loading^34,43^, and that some drugs may be modified to self-assemble^55^. We believe our work builds on these studies by demonstrating this co-assembly for a drug that is unmodified and approved for clinical use. MSNs are an ideal reservoir material for drug release applications, especially when physical strength, consistent and predictable long-term release, and complete access to drug reservoir is desired. Work continues with the material developed here to incorporate OCT-templated and other drug-templated silica nanoparticles into antimicrobial polymeric restorative and implant coating systems, as well as continued investigation of the synthesis kinetics to optimize the co-assembly process. These underlying improvements in the drug-carrier particles could translate into improvements in therapeutic longevity in the prevention of secondary caries over previous antimicrobial MSN-resin composites and implant coatings^25,33,74^.

## Methods

### Chemicals

All chemicals were purchased from commercial sources and used without further purification. Type 1 ultra-pure water was used at 18.2 MΩ cm (Millipore Direct-Q system). Octenidine dihydrochloride (OCT) was purchased from TCI America (Portland, OR, USA). TEOS, ammonium hydroxide (29% weight in water) and CTAB were purchased from Sigma-Aldrich (Oakville, ON, Canada). Sodium hydroxide (10.0 N), hydrochloric acid (6.0 N), sodium chloride, potassium chloride, disodium phosphate, and monopotassium phosphate were purchased from Bioshop Canada Inc. (Burlington, ON, Canada). Anhydrous ethanol was purchased through the University of Toronto MedStore (house brand, Toronto, ON, Canada).

### Mesoporous Silica Nanoparticle preparation

Synthesis of OCT-MSNs was carried out in a 15 mL total volume with the molar ratios 150 H_2_O: 0.052 NaOH: 0.03 OCT: 1 TEOS. OCT was dissolved in water before adding NaOH. Solutions were stirred at approximately 750 RPM using a magnetic stir bar, while TEOS was added drop-wise over 30 s. Concentration of OCT was varied as per the experiment. Solutions were stirred for 30 min before being allowed to age for an additional 23.5 h. Solutions were centrifuged for 1 h at 10 kRPM and supernatant was removed, before adding water, vortexing for 30 s and centrifuging twice more to remove excess reactants. The recovered OCT-MSNs were dried for 24 h at 65 °C before being ground using a mortar and pestle. MCM-41 was produced following an identical procedure replacing OCT with CTAB and using the molar ratios 4000 H_2_O: 30.2 NH_3_: 0.125 CTAB: 1 TEOS. Recovered particles were washed in water using the same procedure as OCT-MSNs, calcined at 550 °C for 6 h, and suspended in OCT dissolved in ethanol for loading. The solutions were mixed and ethanol was allowed to evaporate at 37 °C.

### Critical Micelle Concentration Estimation of Octenidine Dihydrochloride by Conductivity Measurement

OCT was dissolved in ultra-pure water at 5 mM and lowered to 2 mM while monitoring conductivity (Thermo Scientific Orion VERSA STAR with conductivity module and Orion DuraProbe 4-Electrode Conductivity Cell). CMC was estimated as the point of inflection when conductivity was plotted against octenidine concentration.

### MSN Analysis

Electron microscopy was performed using field emission SEM, at 1 kV accelerating voltage (Hitachi SU8230), TEM at 300 kV accelerating voltage with EDX mapping and imaging (Hitachi HF3300), low magnification SEM for overview at 2 kV accelerating voltage (Hitachi SU3500), and TEM for imaging particles for micrographic release tracking study at 200 kV accelerating voltage (FEI Tecnai 20). TEM samples were prepared using ultrathin carbon on holy carbon copper grids. For all electron microscopy imaging techniques, particles were imaged as-is, with no further preparation. XRD was carried out from 1 to 6° using a beam energy of 30 kV and current of 10 mA from a Cu K α radiation source (Rigaku MiniFlex 600). BET analysis was carried out with density functional theory data analysis (Autosorb-1-C and Quantachrome software version 2.11). TGA was carried out using a 10 °C min^−1^ ramp to 550 °C (TA Instruments Q50 TGA). MS identification and confirmation of OCT purity was carried out using ultra high-performance liquid chromatography combined with mass spectrometry (Waters Xevo G2-XS QTof). OCT was dissolved in MS-grade methanol (Thermo Fisher Scientific, Mississauga, ON, Canada) for the pre-synthesis control. OCT-MSNs stored in ambient temperature and humidity for 21 months post synthesis and washing were suspended in MS-grade methanol, allowed to settle, and a fraction was taken and diluted for analysis.

### OCT Release Analysis

MSNs were suspended in PBS prepared via the Cold Spring Harbor protocol and adjusted to a pH of 7.2 through the addition of HCl. OCT concentration in the media was monitored either continuously by fiber-optic probe, or cuvette measurements taken after centrifuging for 1 h at 10 kRPM a 2 mL aliquot from the sample, at λ_max_ = 281 nm (Agilent Cary 60 UV-Vis spectrophotometer). Aliquots were re-suspended and returned to the release sample after reading.

### Release Monitoring by TEM

Samples of OCT-MSNs were suspended in ethanol using a low energy bath sonicator until the desired release of OCT was detected by UV-Vis. Ethanol was chosen to increase drug dissolution and diffusion to induce 100% drug release quickly, as OCT is more soluble in it than in water or biologically relevant buffer. In addition, ethanol will not hydrolize and decompose the silica structure during extraction, thus this inert environment is ideal for prolonged extraction of drug via sonication without altering the particle. Samples were centrifuged for 1 h at 10 kRPM and dried at 65 °C for 24 h before being deposited on ultrathin C supported by holey carbon TEM grids. remaining drug load was confirmed by TGA. Imaging was carried out as before (FEI Tecnai 20). Image analysis was performed (ImageJ with Radial Profile Extended add-on available at https://imagej.nih.gov/ij/plugins/radial-profile-ext.html). Pixel intensity values were taken from a random bisecting line through the centre of 7 different particles from separate images at each of the 5 loading value (35 images, 35 particles total), as well as the radially integrated intensity for the whole particle at each distance r from the particle centre to the maximum radius R. Only areas of the image where the particle was resting on ultrathin carbon with no obstruction were measured. r values were normalized from 0 to R = 1, and pixel intensity values of each image were normalized by the darkest point in each particle’s centre (0) and an average intensity of a random area of background ultrathin carbon film (1). RMS was calculated at each r value across the 7 particles of the same drug loading from the difference between the bisecting line intensity and the radially integrated intensity from each of those particles, thus giving an approximate value describing the porous appearance at any r value for particles of different loading.

### Antimicrobial Analysis

MIC of as-received OCT and OCT released from OCT-MSNs were determined. Stock solutions were prepared by dissolving powdered drug in PBS by sonication and sterilised through a 0.2 µm syringe filter. Additionally, solutions made from released OCT from OCT-MSNs suspended in PBS at 2 mg mL^−1^, filtered via centrifugation, and quantified via UV-vis spectroscopy. *S. mutans* UA 159 was grown to mid-log phase at 37 °C and 5% CO_2_ in Todd Hewitt broth supplemented with 10% yeast extract (THYE, BD Biosciences, Mississauga, ON)^75^. *S. mutans* was diluted 1/20 in 5 mL THYE and incubated for approximately 3 hours. Dilutions of OCT solutions were made in THYE and added in 100 µL quantities to a 96-well plate. At a growth OD of 0.2 cultures were diluted to an OD of 0.1 in THYE and 100 µL was added to the plate with OCT dilutions, with uninoculated THYE as a control. Plates were incubated 24 hours at 37 °C and 5% CO_2_. Growth or no-growth was determined by visual inspection with OD confirmation via microplate reader (Bio-Tek Cytation 3). N = 4 cultures with 3 replicates for all measurements, with a 1 µg mL^−1^ resolution in MIC and consistent results across measurements.

### Data availability statement

The data reported on in this study are available from the corresponding author upon reasonable request.

## Electronic supplementary material

Supplemental Material
