# Supplementary material for: Drug self-assembly for synthesis of highly-loaded antimicrobial drug-silica particles
Source: Sci Rep. 2018 Jan 17;8:895. doi: 10.1038/s41598-018-19166-8 (PMC5772632; doi:10.1038/s41598-018-19166-8)
Supplement: Supplementary file 1 — Supplemental Material [file 41598_2018_19166_MOESM1_ESM.doc]

**Drug self-assembly for synthesis of highly-loaded antimicrobial drug-silica particles**

**Supplementary Material**

Cameron A. Stewart^1^, Yoav Finer^1,2^, Benjamin D. Hatton^1,3^*

1. Institute of Biomaterials and Biomedical Engineering, University of Toronto, Toronto, Ontario, Canada
2. Faculty of Dentistry, University of Toronto, Toronto, Ontario, Canada
3. Department of Materials Science and Engineering, University of Toronto, Toronto, Ontario, Canada


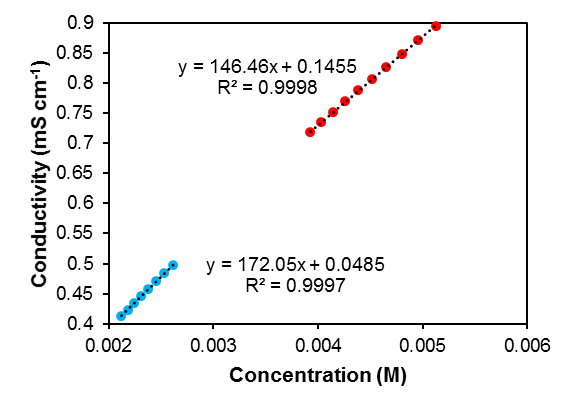
**Supplementary Figure S1:** Conductivity measurements are used to estimate the Critical Micelle Concentration (CMC) of Octenidine Dihydrochloride in ultrapure water as approximately 3.79 mM. The two linear sections of measurements produce different linear regression formulas, and the intersection is interpreted as the CMC. Linear regression formulas and R^2^ values are inset.


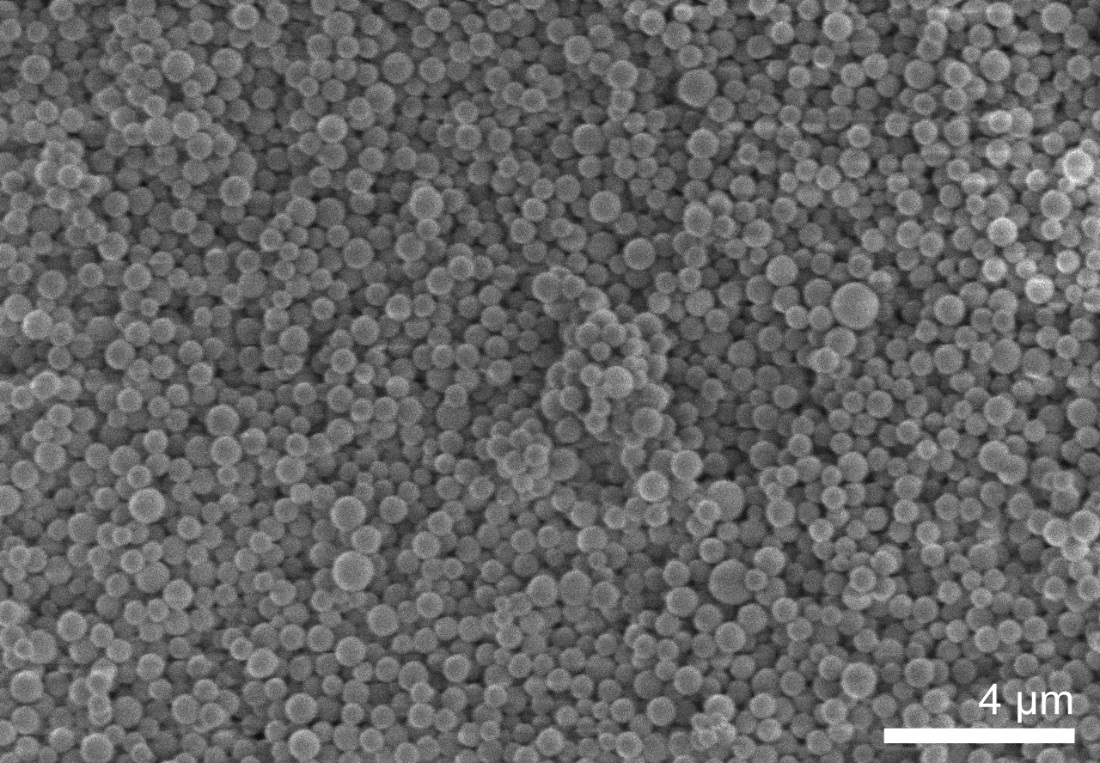


**Supplementary Figure S2:** SEM micrograph of OCT-MSNs demonstrating low polydispersity of size and shape (Hitachi SU3500 SEM, 2 kV accelerating voltage).


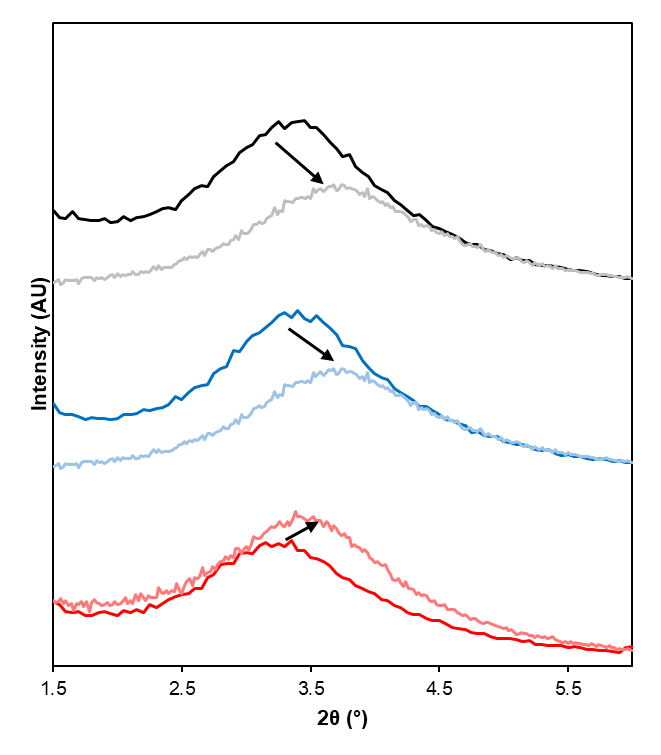


**Supplementary Figure S3:** Calcination of OCT-MSNs shifts XRD peaks. The peak shift (highlighted by arrows) is caused by shrinking of the silica matrix bringing pores closer together, and is consistent with known mesostructure behavior. The permanence of a diffraction peak suggests that the peak is caused by a silica mesostructure, and not crystalized drug. Y axis is XRD intensity in arbitrary units and peak intensity is not comparable.


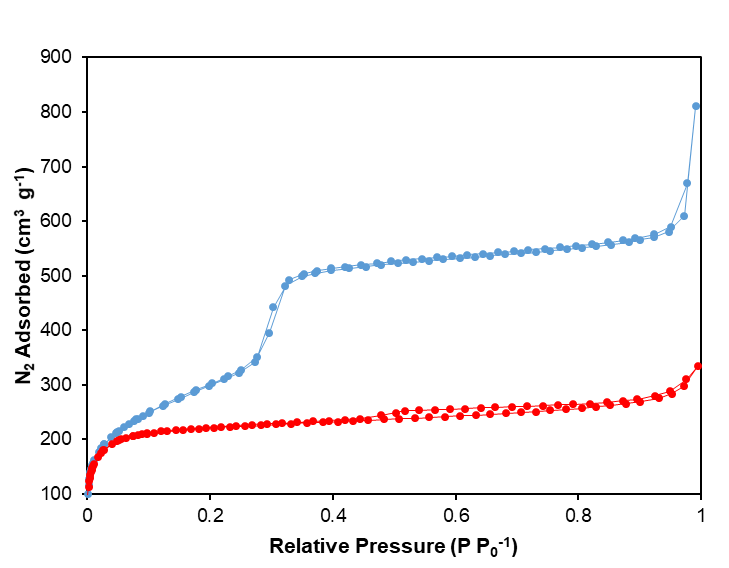


**Supplementary Figure S4:** Raw N_2_ BET adsorption and desorption isotherms of OCT-MSNs (red) and MCM-41 control (blue) after calcining at 550 °C for 6 h. Isotherms are typical of micro- and mesoporous systems, but demonstrate the difference in porosity between OCT-MSNs and a mesoporous structure synthesized with a more typical surfactant pore template.

**Supplementary Figure S5:** TGA is used to determine the internal drug content of OCT-MSNs. Weight change is taken from approximately 250 °C (after water evaporation) to 550 °C and represents the OCT content within MSNs by weight. Displayed is an exemplary TGA curve.


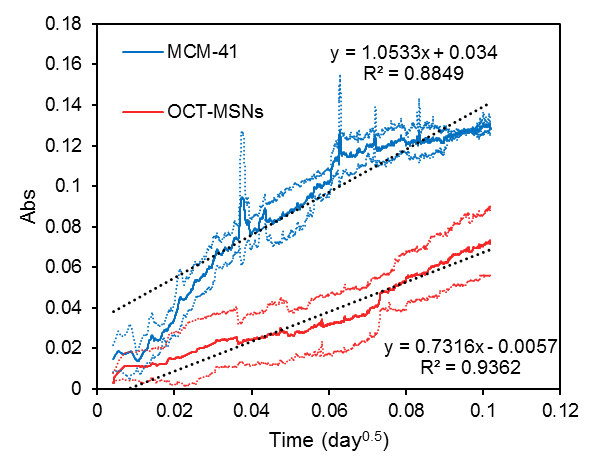


**Supplementary Figure S6:** Absorbance of OCT at 281 nm measured by fibre optic probe released from either control OCT-loaded MCM-41 (blue) or OCT-MSNs (red), plotted against the square-root of time and linear regression results to demonstrate fitting to the Higuchi model of release from a porous material.


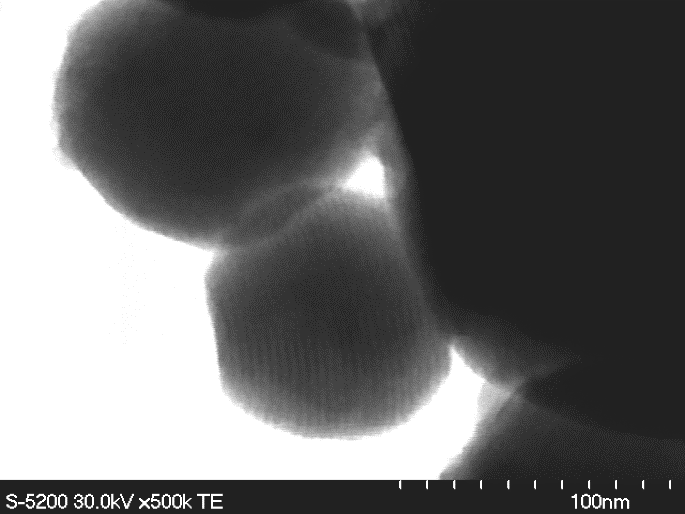

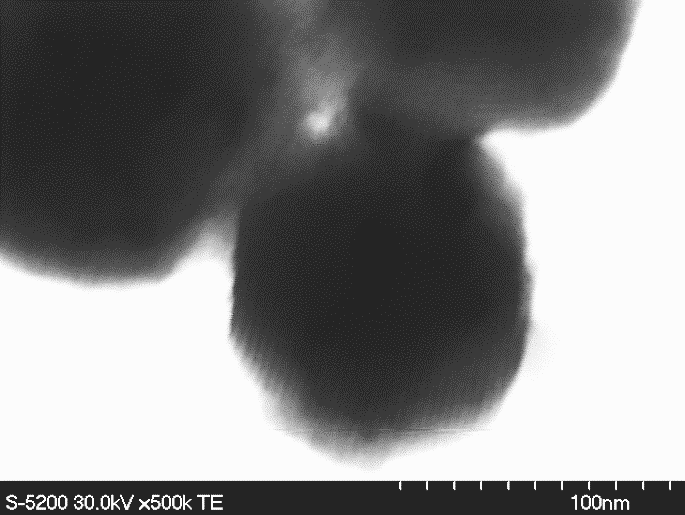


**A**

**B**


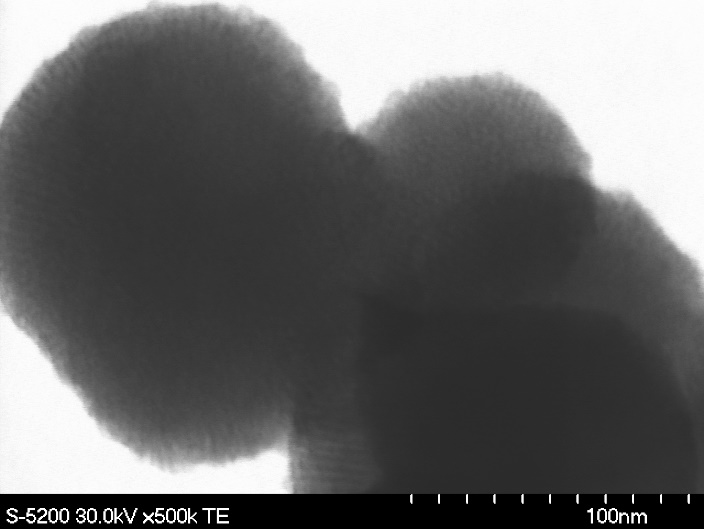

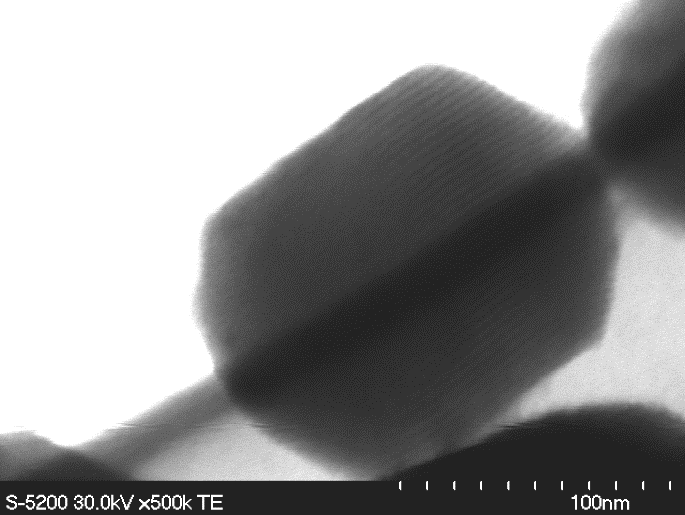


**C**

**D**

**Supplementary Figure S7:** Scanning TEM images of calcined MCM-41 prior to drug loading. No exterior layer is seen around these particles, unlike MCM-41 with loaded OCT shown in the paper text. C demonstrates a top-down fiew of aligned pores, while A, B and D show a side view with the typical contrasting lines running the length of the particles. D is partially obscured by the TEM grid backing. Samples were prepared by depositing dried MCM-41 onto ultrathin C supported by holey carbon TEM grids and blowing away excess material, and imaged using a Hitachi S-5200 high resolution SEM in transmission mode at 30 kV_Acc_.
